# Independent and joint trajectories of depression and anxiety symptoms among Chinese male sailors throughout a prolonged non-24-h rotating shift schedule at sea: a parallel-process growth mixture modeling approach

**DOI:** 10.1186/s12888-023-05389-1

**Published:** 2023-12-11

**Authors:** Zhihao Tu, Fei Tian, Jingwen He, Chuan Wang, Jianquan Tian, Xinghua Shen

**Affiliations:** 1 Navy Medical Center, Naval Medical University, Shanghai, China; 2 Qingdao Special Servicemen Recuperation Center of PLA Navy, Qingdao, China; 3 Department of General Practice, The First Affiliated Hospital of Naval Medical University, Shanghai, China; 4 Shanghai University of Traditional Chinese Medicine, Shanghai, China; 5 Department of Psychology, Naval Medical University, Shanghai, China; 6 Key Laboratory of Molecular Neurobiology of Ministry of Education, Shanghai, China

**Keywords:** Depression, Anxiety, Hardiness, Shift work tolerance, Rotating shift work, Longitudinal study

## Abstract

**Background:**

The predictive and protective effect of hardiness on mental health remains unclear among shift workers on non-24-h working schedules. The present study aimed to investigate the independent and joint trajectories of depression and anxiety symptoms and the role of hardiness during a prolonged period of non-24-h shift working schedule.

**Methods:**

Four hundred nine Chinese male sailors (working on 18-h watchstanding schedule) were recruited and completed all 5-wave tests through online questionnaires (at Day 1, 14, 28, 42, 55, respectively) during a 55-day sailing. The questionnaires included sociodemographic variables, hardiness, depression and anxiety symptoms. Independent and joint trajectories of depression and anxiety symptoms were estimated by latent growth mixture models. The effect of hardiness on trajectories was examined by logistic regression models.

**Results:**

2 and 3 latent trajectories were identified for depression and anxiety symptoms, respectively. Based on initial levels and development trends, 3 distinct joint trajectories of depression and anxiety were identifed and named as: “Low-Inverted U” group (73.6%), “Moderate-Deterioration” group (6.9%), and “High-Stable” group (9.5%). Sailors with higher levels of hardiness were more likely to follow the “Low-Inverted U” trajectory of depression and anxiety symptoms (all *p* < 0.001).

**Conclusions:**

There existed individual differences in the trajectories of depression and anxiety. Hardiness may have a protective effect that can prevent and alleviate depression and anxiety symptoms. Therefore, hardiness-based intervention programs are encouraged among the shift workers on non-24-h working and rest schedules.

## Introduction

It is estimated that 11.6%—16.8% of full-time wage and salary workers are shift workers [1, 2]. Shift workers are more vulnerable to psychopathologies than daytime workers, such as depression and anxiety [3, 4]. For example, according to a recent meta-analysis of longitudinal studies, shift workers had 33% higher risk of depressive symptoms than daytime workers [3]. Mental disorders can further lead to negative outcomes such as more sick leave and family relationship conflicts, greatly affecting the performance and well-being of shift workers [5, 6].

Besides common night shift and rotating shift schedules, a substantial proportion of shift workers, such as sailors, submariners, and astronauts, are required to live and work in more special non-24-h working and rest schedules than night shift workers or 24-h rotating shift workers [7–9]. For instance, part of U.S. naval sailors follow a rotating watchstanding schedule with 5-h on and 10-h off (5 h for working and 10 h for leisure and sleep), and all U.S. naval submariners follow an 18-h schedule (6-h on and 12-h off) [10, 11]. Different from regular shift workers, shift workers on non-24-h working schedules have to follow this special regimen for more than 2 months without break. During this period, shift workers are exposed to stressful environment and lack of adequate medical support [12]. Previous studies suggested that submarine crews experienced less positive mood during the patrol period than they did onshore [13, 14]. Thus, considering the negative outcomes of depression and anxiety, it is important to investigate the trajectory of depression and anxiety during the non-24-h regimen.

A few longitudinal studies reported changes of shift workers’ mood state and mental health status during the non-24-h regimen [8, 13, 15], but results were mixed. Some studies showed that submariners and sailors reported more depression and anxiety symptoms after non-24-h working conditions than they reported onshore with normal daily routines [13]. In contrast, other studies suggested that there were no statistically significant changes of submariners’ mood states during the patrol missions [8, 15]. Such population-level inconsistency may result from different watchstanding schedules, mission durations, and living environments in these studies [10, 16–18]. Another possible explanation for such inconsistency is that the shift workers’ mental health status were measured at different stages of prolonged missions. For example, a meta-analysis of 6 studies on mental health status of Chinese submariners before and after patrol missions (measured onshore at 48 h before and after mission) found that submariners reported more depression and anxiety sypmtoms after patrol missions. However, Trousselard et al. [8] found that French submariners did not show statistically significant changes between mood states measured at Day 21 and Day 51 during a 70-day patrol mission at sea. In addition, there were too few measurement time points to capture the true trajectory of shift workers’ mental health status during non-24-h regimen in previous studies. Most of previous studies only employed 2 measurement time points (e.g., pre-mission v.s. post-mission, or beginning of mission v.s. end of mission), neglecting the changing trend in the middle of the whole non-24-h regimen [8, 15]. However, detailed changing trend of depression and anxiety during non-24-h regimen is of great significance for the timing of psychological intervention. What is more, few studies paid attention to the co-existence of depression and anxiety symptoms in the study field of shift work, although numerous studies have shown that the comorbid states of depression and anxiety are common [19–21]. Thus, it is important to conduct a multiple-wave longitudinal study focusing on the independent and joint trajectories of depression and anxiety among shift workers during a period of non-24-h working schedule.

In addition to the aforementioned population-level factors, individual-level factors may also contribute to unique changes in developmental trends of mental health status during shift work. Researchers used the term “shift work tolerance” to describe the individual variability in the adaptability to shift work [22, 23]. Shift workers with less self-reported shift-work-related problems (e.g. sleepiness, fatigue, insomnia, depression, anxiety, digestive problems) were regarded as having higher shift work tolerance [22, 24]. It is of great significance to identify predictors of shift work tolerance for personnel selection (e.g., submariners, navy sailors, astronauts) and shift-work-related problems intervening in the future. Previous studies found that age, gender, chronotype, personality, and certain genetic dispositions were predictors of shift work tolerance [22, 23]. According to the recent influential review, hardiness is most consistently associated with shift work tolerance among all predictors in published studies [22]. Hardiness has been widely recognized as a resource for promoting resilience and against the development of illnesses in the face of stressful situations [24, 25]. A study on female Norwegian nurses working rotating shifts showed that hardiness predicted depression and anxiety after 1-year rotating shift work [24]. Similarly, another study on Norwegian nurses employed in shift work found that subfactors of hardiness predicted depression and anxiety over 2 years [26]. According to the job demands-resources model, hardiness can be treated as personal recources, which means that those who have higher level of hardiness are less likely to have mental disorders caused by work stress [27, 28]. However, no studies investigated the effect of hardiness on developmental trends of depression and anxiety during non-24-h working schedule.

Therefore, the present study aimed to examine (1) the independent and joint trajectories of depression and anxiety symptoms among at-sea Chinese sailors on 18-h watchstanding schedule (6-h on and 12-h off) during a 55-day sailing; (2) the role of hardiness in predicting the trajectories. We postulated that (1) there would exist at least 2 different groups of sailors with different independent and joint trajectories of depression and anxiety symptoms. One subgroup would keep low levels of depression and anxiety throught out the period of non-24-h rotating shift schedule (with slight fluctuation), while another subgroup would present a continuous increase in anxiety and depression symptoms during the period; (2) the baseline level of hardiness would predict subgroup membership of these trajectories: those with higher level of hardiness would have less depression and anxiety symptoms during the period of non-24-h rotating shift schedule.

## Methods

### Participants and procedure

We conducted cluster sampling on Chinese civilian sailors working 18-h watchstanding schedule (6-h on and 12-h off) from 3 ships. The selected 3 ships belonged to the same fleet, having the same ship type and sailing the same route. As there were no female sailors on these ships, all participants were male sailors in the present study. Participants were invited to complete an online questionnaire including sociodemographic variables (i.e., age, marital status, education, only child or not), hardiness, depression, and anxiety using Wenjuanxing (https://www.wjx.cn, a widely used smartphone-based online questionnaire data collection instrument in China). The whole sailing last for 55 days from September 2021 to October 2021. The 3 ships did not dock at any port and all participants kept the 18-h watchstanding schedule during the entire voyage. We set 5 measurement time points throughout the sailing: (1) T1: Day 1 of sailing; (2) T2: Day 14 of sailing; (3) T3: Day 28 of sailing; (4) T4: Day 42 of sailing; (5) T5: Day 55 of sailing.

The study procedures were carried out in accordance with the Declaration of Helsinki. The Institutional Review Board of the Naval Medical University approved the study protocol. All subjects were provided written informed consent. Participants received a compensation of 100 CNY (approximately 14 USD) for their participation. With the aid of Wenjuanxing, participants were required to complete all items before submission, thus there were no missing data. Of the 434 sailors who completed the questionnaire at T1, 421 (94.7%), 415 (95.6%), 413 (95.2%), and 409 (94.2%) participants completed the questionnaire at T2, T3, T4, T5, respectively. Finally, the data of participants who completed the qustionnaires at all measurement time point were included in the formal analyses. Finally, the data of participants were identified by their mobilephone numbers during the data collection and processing. An a-priori power analysis was conducted using G*Power 3.1.9.7 [29] with the parameters recommended by Liang et al. [30]: F-test, ANOVA repeated measures, effect size *f* = 0.1 (small effect size), α = 0.05, statistical power = 0.95, number of groups = 5, number of measurements = 5, correlation among repeated measures = 0.5, and non-sphericity correction *ε* = 0.99 [31]. The result showed that a sample size of *n* = 190 was necessary, and therefore the minimum sample size (*n* = 409) of the 5 waves were enough for analyzing the latent trajectories [31].

### Measures

#### Sociodemographic variables

Participants were asked to report their age (years), marital status (married or single/ divorced/widowed), education (years), only child or not, shift work exposure (years of being a sailor on 18-h working schedule).

#### Hardiness

Hardiness was measured by the Chinese version of 15-item Dispositional Resilience Scale (C-DRS-15) [32]. The original version of Dispositional Resilience Scale was developed by Barton [33] and the C-DRS-15 was translated and validated by Wong et al. (2014). The C-DRS-15 includes 3 subscales: commitment, control, and challenge [32, 33].The item responses are on a 4-point Likert scale ranging from 0 (‘‘not at all true’’) to 3 (‘‘completely true’’), generating total scores from 0 to 45. A higher score represented greater psychological hardiness. In this study, the internal consistency of C-DRS-15 was acceptable: Cronbach’s *α* = 0.79.

#### Depression symptoms

The depression symptoms in the last 2 week were assessed by the 20-item Zung Self-rating Depression Scale (SDS**)** [34]*.* The item responses are on a 4-point Likert scale ranging from 1 (‘‘none or a little of the time’’) to 4 (‘‘most or all of the time’’), generating raw score from 20 to 80. Then, the raw score was multiplied by 1.25 and rounded to create the standard score ranging from 25 to 100, which was regarded as total score of SDS. A higher score represented greater depression symptoms. The Chinese version of SDS was used in this study [35]. The reliability and validity of SDS were demonstrated in Chinese samples [35, 36]. The Cronbach’s α of SDS at 5 time points in the present study were 0.84, 0.83, 0.85, 0.85, and 0.86, respectively.

#### Anxiety symptoms

The anxiety symptoms in the last 2 week were assessed by the 20-item Zung Self-rating Anxiety Scale (SAS) [37]. The item responses are on a 4-point Likert scale ranging from 1 (‘‘none or a little of the time’’) to 4 (‘‘most or all of the time’’), generating raw score from 20 to 80. Then, the raw score was multiplied by 1.25 and rounded to create the standard score ranging from 25 to 100, which was regarded as total score of SAS. A higher score represented greater anxiety symptoms. The Chinese version of SAS was used in this study [38]. The relaibility and validity of SAS were demonstrated in Chinese samples [39, 40]. In this study, the Cronbach’s α of SAS at 5 time points were 0.88, 0.87, 0.89, 0.88, and 0.88, respectively.

### Data analysis

Statistical analyses were conducted using R software (version 4.2.2) for Windows. Latent growth mixture models (LGMM) were conducted by *Mplus* Version 8.3.

Before formal analyses, we used χ^2^ tests and independent samples *t* tests to compare categorical and continuous variables of participants in analytical samples and excluded samples (the participants who did not complete all 5-wave measurements and thus were not included in the final analysis), examining whether there existed significant differences between analytical samples and excluded samples.

Then, LGMM were used to measure heterogeneity of depression and anxiety symptoms over time throughout the sailing [41]. The analyses of LGCM described in this study were conducted in the following 3 steps:

- 1. Latent curve growth analysis (LCGA) were established to determine if a linear or quadratic trend fit the overall sample trajectory better [30]. Factor loadings of the time points were set as 0, 1, 2, 3, 4 given the interval between measurement points was equal (2 weeks between T1, T2, T3, T4, and T5) [42]. For single LCGA, model fit was accessed by the Tucker-Lewis index (TLI), comparative fit index (CFI), root mean square error of approximation (RMSEA), and standardized root mean square residual (SRMR) [43, 44]. According to Hu and Bentler [45], TLI ≥ 0.90, CFI ≥ 0.90, RMSEA ≤ 0.08, and SRMR < 0.05 were considered to indicate acceptable model fit.
- 2. LGMM were established for depression and anxiety symptoms separately. We run 1 to 5 class solutions of LGMM sequentially to determine the optimal number of latent classes [46, 47]. The intercept and slope variance parameters were allowed to vary within classes [42]. The optimal number was determined by considering a series of fit statistics, including Bayesian information criteria (BIC), sample-size adjusted Bayesian information criteria (aBIC), Aikaike information criterion (AIC), entropy values, the Lo-Mendell-Rubin likelihood ratio test (LRT), and the bootstrap likelihood ratio test (BLRT). A better model fit was indicated by lower information criteria indices (BIC, AIC, aBIC), higher entropy value, a signifcant LRT and BLRT results [30, 42]. The final decision on the number of latent classes were made based on fit indices and theoretical interpretability [42]. The LGMM were estimated using robust maximum likelihood method. The number of random start values and final iterations included were set as 1000 and 120 respectively [41, 42].
- 3. Finally, we used standard three-step method to examine whether sociodemographic variables and hardiness can predict class membership [41]. The classification results of the final selected LGMM were extracted from Mplus. Then, multinomial logistic regression analyses were condected using sociodemographic variables and hardiness as independent variables and class membership as dependent variable [30]. Odds ratios with a 95% confdence interval (CI) were reported. A certain independent variable was regarded as a predictor for subgroup membership of certain trajectories, if its 95% CI odds ratio did not contain 1.

Joint trajectories of depression and anxiety symptoms were examined using parallel process lantent growth mixture models (PPLGMM). The procedure of PPLGMM was similar to that of LGMM mentioned above [30, 48–50].

## Results

### Characteristics of participants

Table 1 showed the baseline sociodemographic variables, hardiness, depresion, and anxiety of the analytical samples and excluded samples. There were no statistically signifcant differences between variables of the two samples. The mean age of the final analytical sample was 30.44 years (SD = 6.13). The mean score (SD) of hardiness at T1 was 32.72 (4.66). The mean scores (SD) of SDS scores at T1, T2, T3, T4, and T5 were 37.57 (7.38), 40.66 (7.69), 37.65 (7.07), 38.45 (7.61), and 38.10 (7.93), respectively. The mean scores (SD) of SAS scores at T1, T2, T3, T4, and T5 were 41.92 (9.58), 44.68 (8.57), 44.39 (9.74), 44.97 (9.21), and 42.32 (9.59), respectively.

**Table 1:** Comparison of baseline variables between the analytical sample and the excluded sample

Variables	Analytical sample		Excluded sample		*p*
	N/mean	%/SD	N/mean	%/SD	
Marital status					0.784
Married	234	57.21	15	60.00	
Single/divorced/widowed	175	42.79	10	40.00	
Siblings					0.998
Single	131	32.03	8	32.00	
Non-single	278	67.97	17	68.00	
Age (years)	30.44	6.13	29.13	5.33	0.297
Education (years)	11.98	2.89	12.17	3.42	0.752
Shift work exposure (years)	9.32	5.42	10.39	5.16	0.337
Hardiness	32.72	4.66	31.99	4.21	0.445
Depression	37.57	7.38	38.54	7.89	0.525
Anxiety	41.92	9.58	42.61	9.56	0.727

### Independent trajectories of depression and anxiety symptoms

Table 2 showed the zero order correlations of sociodemographic variables (age, marital status, education, only child or not, shift work exposure), hardiness, depression and anxiety at Day 1 of sailing (T1), Day 14 of sailing (T2), Day 28 of sailing (T3), Day 42 of sailing (T4), Day 55 of sailing (T5).

**Table 2:** Zero order correlations of demographic variables, hardiness, depression and anxiety at T1, T2, T3, T4, T5

	1	2	3	4	5	6	7	8	9	10	11	12	13	14	15	16
1. Age																
2. Education	.04															
3. Marriage	- .27	- .11														
4. Siblings	.25	.05	- .08													
5. Exposure	.93^***^	- .03	- .18	.19												
6. C-DRS-15	- .04	.19	- .15	.05	- .06											
7. SDS-T1	- .06	.00	.18	- .28^*^	.03	- .48^***^										
8. SDS-T2	- .04	- .10	.23	- .25	.07	- .47^***^	.52^***^									
9. SDS-T3	- .14	- .09	.09	- .22	- .05	- .30^**^	.55^***^	.59^***^								
1. SDS-T4	- .06	- .02	.08	- .31^*^	- .02	- .34^**^	.65^***^	.62^***^	.69^***^							
11. SDS-T5	- .02	.04	.11	- .28^*^	.01	- .41^**^	.64^***^	.45^***^	.60^***^	.60^***^						
12. SAS-T1	- .20	- .00	.13	- .14	- .15	- .26^**^	.65^***^	.49^***^	.62^***^	.60^***^	.40^**^					
13. SAS-T2	- .10	.03	.11	- .14	- .04	- .34^**^	.46^***^	.61^***^	.52^***^	.55^***^	.41^**^	.49^***^				
14. SAS-T3	- .01	- .09	.10	- .04	.04	- .28^**^	.37^**^	.31^***^	.59^***^	.50^***^	.43^**^	.45^***^	.50^***^			
15. SAS-T4	- .02	.03	- .06	- .12	.02	- .29^**^	.38^**^	.41^***^	.53^***^	.59^***^	.47^***^	.48^***^	.64^***^	.71^***^		
16. SAS-T5	.01	.05	- .15	- .19	.02	- .33^**^	.52^***^	.32^*^	.50^***^	.46^***^	.75^***^	.42^**^	.39^**^	.45^***^	.51^***^	

Zero order correlations for longitudinal data (*n* = 409). *Exposure* Shift work exposure, *C-DRS-15* Chinese version of 15-item Dispositional Resilience Scale, *SDS* Zung Self-rating Depression Scale, *SAS* Zung Self-rating Anxiety Scale. Pearson’s correlation (two-tailed)

^*^ *p* < .05. ^**^ *p* < .01. ^***^ *p* < .001

The model-fit results of independent LGCM for depression and anxiety were presented in Table 3. For depression, the model fit of quadratic curvilinear model (CFI = 0.94, TLI = 0.93, RMSEA = 0.07, SRMR = 0.04) was better than that of single linear model (CFI = 0.91, TLI = 0.89, RMSEA = 0.06, SRMR = 0.05). The intercept (*I*), slope (*S*), and quadratic (*Q*) of the quadratic curvilinear model were: (1) *I* = 37.73, *p* < 0.001; (2) *S* = 1.49, *p* < 0.001; (3) *Q* = -0.32, *p* < 0.001. Thus, quadratic term should be included in the LGMM of depression. Similarly, for anxiety, the model fit of quadratic curvilinear model (CFI = 0.96, TLI = 0.92, RMSEA = 0.06, SRMR = 0.04) was much better than that of single linear model (CFI = 0.69, TLI = 0.69, RMSEA = 0.26, SRMR = 0.21), with (1) *I* = 41.67, *p* < 0.001; (2) *S* = 3.72, *p* < 0.001; (3) *Q* = -0.90, *p* < 0.001. Thus, quadratic term should be included in the LGMM of anxiety.

**Table 3:** Single-Factor Model Goodness-of-Fit Indexes of LGCM for depression and anxiety

Model	*χ* ^2^ (*df*)	CFI	TLI	RMSEA [90% CI]	SRMR
Depression: Linear	119.44(10)^***^	.91	.89	.06 [.03, .09]	.05
Depression: Linear + Quadratic	91.70(6)^***^	.94	.93	.07 [.04, .10]	.04
Anxiety: Linear	287.48(10)^***^	.69	.69	.26 [.23, .28]	.21
Anxiety: Linear + Quadratic	57.97(6)^***^	.96	.92	.06 [.03, .09]	.04

*Df* Degrees of freedom, *CFI* Comparative fit index, *TLI* Tucker–Lewis index, *RMSEA* Root mean squared error of approximation, *CI* Confidence interval, *SRMR* Standardized root mean square residual

^***^ *p* < .001

**Fig. 1:**
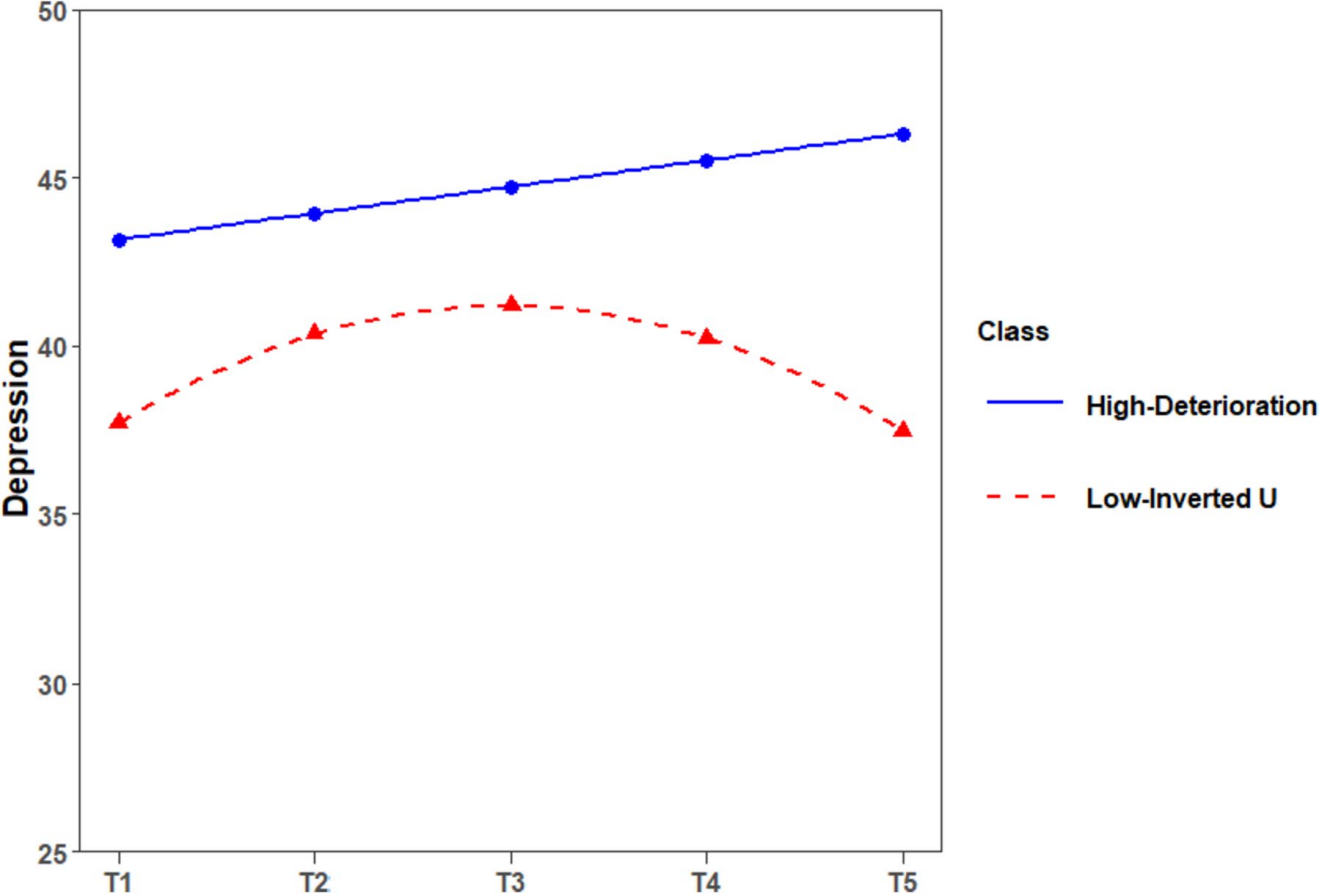
The estimated latent trajectories of depression symptoms

A comparison of 5 models suggested that a 2-class curvilinear model provided the best fit for depression (see Table 4). The 3-class solution demonstrated a nonsignificant result of LMR test, indicating that 3 classes did not fit better than 2 classes did. Thus, considering the model parsimony, 2-class model was better than 3-class model. For the same reason, 2-class model was better than 4-class and 5-class model for depression. The depression symptom trajectories for 2-class solution were presented in Fig. 1. Based on initial levels and development trends, the 2 classes were named as “High-Deterioration” group (*n* = 100, 24.5%) and “Low-Inverted U” group (*n* = 309, 75.5%). The High-Deterioration group followed a linear growth trend with a higher intercept (*I* = 43.15, *p* < 0.001), a gentle linear slope (*S* = 0.79, *p* < 0.001), but a non-significant quadratic term (*Q* = -0.06, *p* = 0.325). The Low-Inverted U group followed an Inverted-U curvilinear trend with a lower intercept (*I* = 37.70, *p* < 0.001), a significant linear slope (*S* = 3.54, *p* < 0.001), and a significant quadratic term (*Q* = -0.90, *p* < 0.001). The High-Deterioration group was characterized by a higher initial level of depression symptoms and a moderate level of growth throughout the entire sailing. The Low-Inverted U group showed a moderate growth during the first half of the sailing, and then a moderate decrease during the second half of the sailing. The mean level of depression symptoms reached its peak value (41.18) at the midpoint (Day 28 of the 55-day sailing) based on the estimated results. Throughout the entire period of non-24-h rotating shift schedule, the average depression level of the Low-Inverted U group at all measurement time point was less than The High-Deterioration group. Therefore, Low-Inverted U group can be treated as a more mentally healthy subgroup than the High-Deterioration group.

**Table 4:** Fit indices for independent trajectories of depression and anxiety symptoms

Model	AIC	BIC	aBIC	Entropy	LMR (*p*)	BLRT (*p*)	Class count and proportions/N (%)
Depression							
**2C**	**14,247.06**	**14,319.95**	**14,262.83**	**0.92**	** < 0.001**	** < 0.001**	**100 (24.5)/309 (75.5)**
3C	13,892.98	13,982.07	13,912.25	0.92	0.052	< 0.001	31 (7.6)/309 (75.6)/69 (16.8)
4C	13,260.68	13,365.04	13,282.54	0.93	0.063	< 0.001	31 (7.6)/309 (75.6)/23 (5.6)/46 (11.2)
5C	13,180.97	13,301.38	13,206.18	0.89	0.079	0.006	255 (62.3)/54 (13.2)/16 (3.9)/31 (7.6)/53 (13.0)
Anxiety							
2C	12,730.52	12,802.77	12,745.65	0.93	< 0.001	< 0.001	69 (16.9)/340 (83.1)
**3C**	**12,672.83**	**12,761.134**	**12,691.32**	**0.95**	**0.016**	** < 0.001**	**77 (18.8)/24 (5.9)/308 (75.3)**
4C	12,618.25	12,722.61	12,640.10	0.95	0.083	< 0.001	308 (75.3)/8 (2.0)/24 (5.9)/69 (16.8)
5C	12,612.60	12,733.01	12,637.81	0.93	0.062	< 0.001	47 (11.5)/16 (3.9)/308 (75.3)/8 (1.9)/30 (7.4)

The final extracted model is bold

**Fig. 2:**
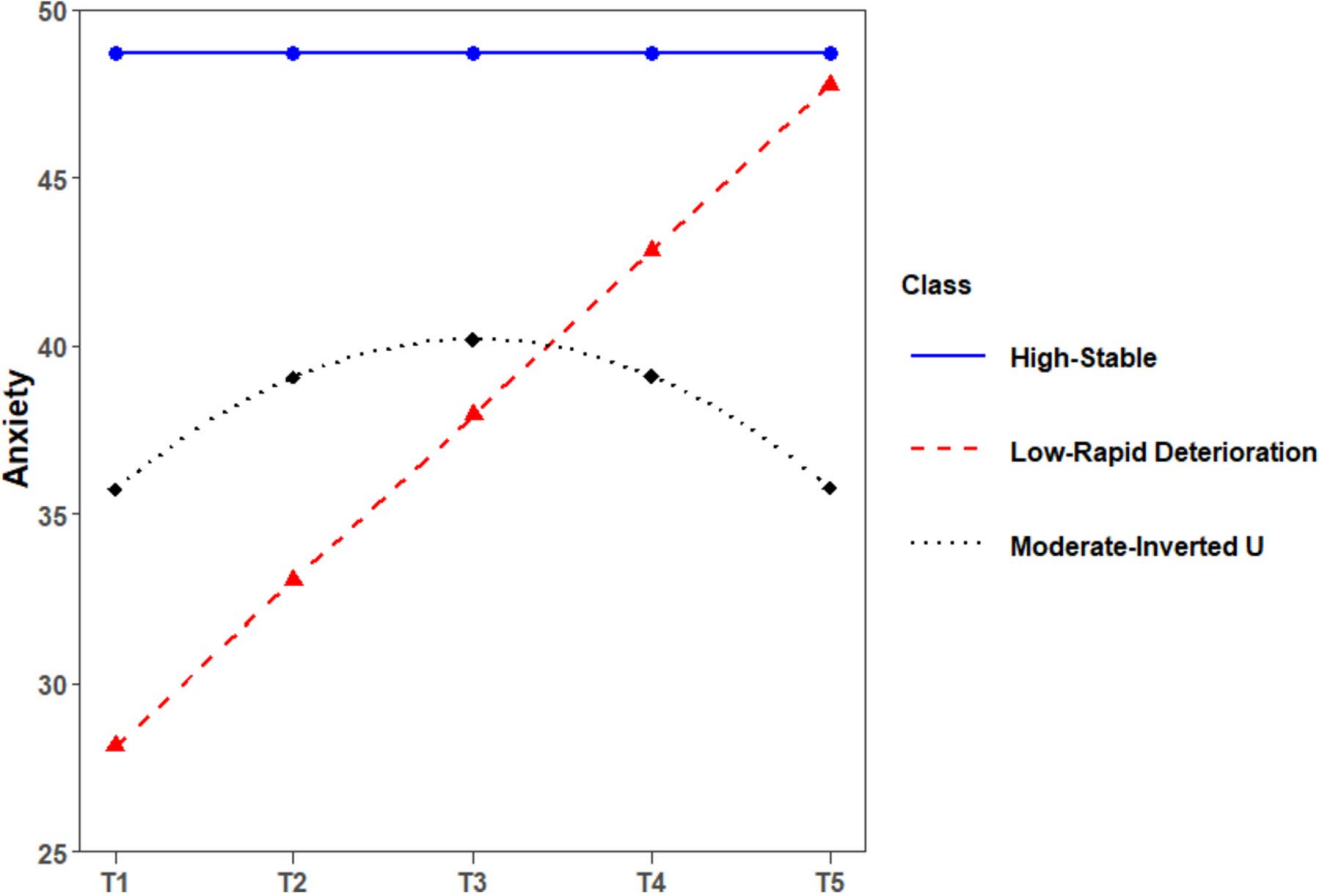
The estimated latent trajectories of anxiety symptoms

 A comparison of 5 models suggested that a 3-class curvilinear model provided the best fit for anxiety (see Table 4). The 3-class solution demonstrated a significant result of LMR and BLRT test, indicating that 3 classes fit better than 2 classes did. But, the 4-class solution did not showed a better fit than the 3-class solution did with a non-significant LMR test (see Table 4). Thus, considering the model parsimony, 3-class model was better than 4-class model. For the same reason, 3-class model was better than 5-class model for anxiety. The anxiety symptom trajectories for 3-class solution were presented in Fig. 2. Based on initial levels and development trends, the 3 classes were named as “High-Stable” group (*n* = 77, 18.8%), “Low-Rapid Deterioration” group (*n* = 24, 5.9%), and “Moderate-Inverted U” group (*n* = 308, 75.3%). The High-Deterioration group kept stable with a higher intercept (*I* = 48.72, *p* < 0.001), a non-significant linear slope (*S* = -0.49, *p* = 0.611), and a non-significant quadratic term (*Q* = -0.01, *p* = 0.958). The Moderate-Inverted U group followed an Inverted-U curvilinear trend with a moderate intercept (*I* = 35.75, *p* < 0.001), a significant linear slope (*S* = 4.45, *p* < 0.001), and a significant quadratic term (*Q* = -1.11, *p* < 0.001). The Low-Rapid Deterioration group followed a rapid growth trend with a low intercept (*I* = 28.12, *p* < 0.001), a steep linear slope (*S* = 4.45, *p* < 0.001), and a non-significant quadratic term (*Q* = -0.19, *p* = 0.545). The High-Stable group was characterized by a higher initial level of anxiety symptoms and a stable level of anxiety throughout the entire sailing. The Moderate-Inverted U group showed a moderate growth during the first half of the sailing, and then a moderate decrease during the second half of the sailing. The mean level of depression symptoms reached its peak value (40.19) at the midpoint (Day 29 of the 55-day sailing) based on the estimated results. The Low-Rapid Deterioration group was characterized by a very low initial level of anxiety symptoms but a rapid growth throughout the entire sailing. Throughout the entire period of non-24-h rotating shift schedule, the average anxiety level of the High-Stable group at all measurement time point was worse than other two groups. Therefore, High-Stable group can be treated as the least mentally healthy subgroup. In additioon, the highest average anxiety level of the Low-Rapid Deterioration group, closed to the clinical cut-off point of SAS, was much worse than that of The Moderate-Inverted U group. Thus, The Moderate-Inverted U group can be treated as the most mentally healthy subgroup among all three subgroups.

Table 5 presented the longitudinal association between baseline hardiness and the independent trajectories of depression symptoms, controlling sociodemographic variables. The Low-Inverted U group was set as reference. For depression symptoms, the results of logistic regression showed that the sailors with higher level of baseline hardiness were less likely to belong to the High-Deterioration group (*OR* = 0.55 [0.47, 0.63], *p* < 0.001). In addition, having siblings (*OR* = 0.19 [0.09, 0.36], *p* < 0.001), more education years(*OR* = 1.40 [1.18, 1.69], *p* < 0.001), and longer shift work exposure (*OR* = 1.23 [1.03, 1.49], *p* = 0.031) were associated with higher likelihood of belonging to the High-Deterioration group.

**Table 5:** The predictive role of hardiness on independent trajectories of depression symptoms

	*B* (*SE*)	*OR* [95% CI]
Married (Other as reference)	0.08 (0.45)	1.09 [0.44, 2.60]
Only child (non-single as reference)	-1.67 (0.34)	0.19 [0.09, 0.36]^***^
Age	-0.14 (0.11)	0.87 [0.70, 1.07]
Education	0.34 (0.09)	1.40 [1.18, 1.69]^***^
Shift work exposure	0.21 (0.09)	1.23 [1.03, 1.49]^*^
Hardiness	-0.60 (0.07)	0.55 [0.47, 0.63]^***^

Low-Inverted U group as reference. Hardiness was standardized before included in the logistic regression model

^*^ *p* < .05. ^**^ *p* < .01. ^***^ *p* < .001

Table 6 presented the longitudinal association between baseline hardiness and the independent trajectories of anxiety symptoms, controlling sociodemographic variables. The Moderate-Inverted U group was set as reference. For anxiety symptoms, the results of multinomial logistic regression showed that the sailors with higher level of baseline hardiness were less likely to belong to the Low-Rapid Deterioration group (*OR* = 0.84 [0.79, 0.89], *p* < 0.001) and High Stable group (*OR* = 0.80 [0.72, 0.88], *p* < 0.001). In addition, less education years (*OR* = 2.04 [1.35, 3.09], *p* < 0.001) were associated with lower likelihood of belonging to the High-Stable group. Besides, the sailors with less education years (*OR* = 1.75 [1.41, 2.16], *p* < 0.001) were also less likely to belong to the High-Stable group.

**Table 6:** The predictive role of hardiness on independent trajectories of anxiety symptoms

	*Low-Rapid Deterioration*		*High-Stable*	
	*B* (*SE*)	*OR* [95% CI]	*B* (*SE*)	*OR* [95% CI]
Married (Other as reference)	-0.45 (0.47)	0.63 [0.29, 1.39]	-0.63 (0.47)	0.53 [0.21, 1.34]
Only child (non-single as reference)	0.51 (0.53)	1.67 [0.60, 4.70]	-0.08 (0.30)	0.93 [0.52, 1.66]
Age	-0.01 (0.14)	0.99 [0.75, 1.31]	-0.17 (0.09)	0.85 [0.70, 1.02]
Education	0.72 (0.21)	2.04 [1.35, 3.09]^***^	0.56 (0.11)	1.75 [1.41, 2.16]^***^
Shift work exposure	-0.19 (0.13)	0.82 [0.64, 1.07]	0.08 (0.08)	1.09 [0.93, 1.27]
Hardiness	-0.18 (0.03)	0.84 [0.79, 0.89]^***^	-0.23 (0.05)	0.80 [0.72, 0.88]^***^

Moderate-Inverted U group as reference. Hardiness was standardized before included in the logistic regression model

^*^ *p* < .05. ^**^ *p* < .01. ^***^ *p* < .001

### Joint trajectories of depression and anxiety symptoms

**Fig. 3:**
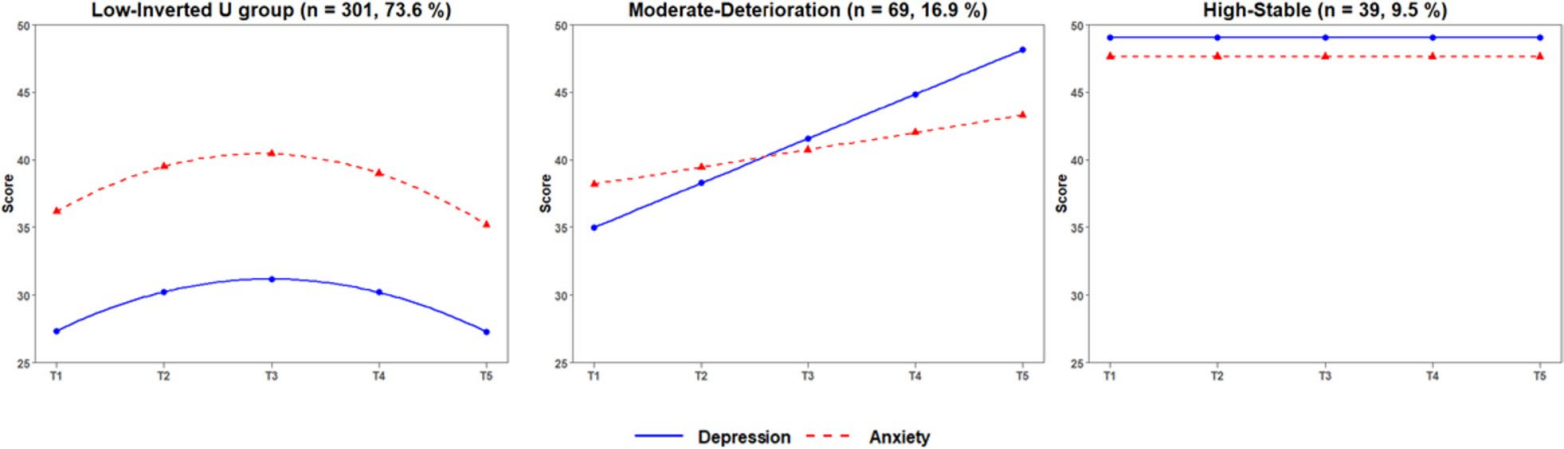
The estimated joint latent trajectories of depression and anxiety symptoms

A comparison of 5 models suggested that a 3-class model provided the best fit for the joint trajectories of depression and anxiety symptoms (see Table 7). The 3-class solution demonstrated a significant result of LMR and BLRT test, indicating that 3 classes fit better than 2 classes did. But, the 4-class solution did not showed a better fit than the 3-class solution did with a non-significant LMR and BLRT test (see Table 7). Thus, considering the model parsimony, 3-class model was better than 4-class model. For the same reason, 3-class model was better than 5-class model. The joint trajectories of depression and anxiety for 3-class solution were presented in Fig. 3. Based on initial levels and development trends, the 3 classes were named as “Low-Inverted U” group (*n* = 301, 73.6%), “Moderate-Deterioration” group (*n* = 69, 16.9%), and “High-Stable” group (*n* = 39, 9.5%). The High-Stable group kept stable with a higher intercept (*I* = 49.05, *p* < 0.001), a non-significant linear slope (*S* = 0.36, *p* = 0.098), and a non-significant quadratic term (*Q* = -0.01, *p* = 0.735) for depression, while a high intercept (*I* = 47.64, *p* < 0.001), a non-significant linear slope (*S* = 0.45, *p* = 0.317), and a non-significant quadratic term (*Q* = -0.11, *p* = 0.088) for anxiety. The Low-Inverted U group followed an Inverted-U curvilinear trend with a low intercept (*I* = 27.35, *p* < 0.001), a significant linear slope (*S* = 3.86, *p* < 0.001), and a significant quadratic term (*Q* = -0.97, *p* < 0.001) for depression, while a low intercept (*I* = 36.17, *p* < 0.001), a significant linear slope (*S* = 4.51, *p* < 0.001), and a significant quadratic term (*Q* = -1.19, *p* < 0.001) for anxiety. The Moderate-Deterioration group followed a linear growth trend with a moderate intercept (*I* = 34.99, *p* < 0.001), a moderate linear slope (*S* = 3.29, *p* < 0.001), and a non-significant quadratic term (*Q* = -0.13, *p* = 0.624) for depression, while a moderate intercept (*I* = 38.17, *p* < 0.001), a gentle linear slope (*S* = 1.28, *p* < 0.001), and a non-significant quadratic term (*Q* = -0.15, *p* = 0.893) for anxiety.

**Table 7:** Fit indices for joint trajectories of depression and anxiety symptoms

Model	AIC	BIC	aBIC	Entropy	LMR (*p*)	BLRT (*p*)	Class count and proportions/N (%)
2C	25,894.12	26,070.72	25,931.10	0.95	< 0.001	< 0.001	108 (26.4)/301 (73.6)
**3C**	**25,528.99**	**25,733.69**	**25,571.85**	**0.97**	** < 0.001**	** < 0.001**	**301 (73.6)/69 (16.9)/39 (9.5)**
4C	25,407.87	25,640.66	25,456.62	0.96	0.063	0.178	301 (73.6)/53 (13.0)/39 (9.5)/16 (3.9)
5C	25,255.94	25,516.83	25,310.57	0.94	0.438	0.524	45 (11.0)/239 (58.4)/62 (15.2)/24 (5.9)/39 (9.5)

The final extracted model is bold

The High-Stable group was characterized by a higher initial level of both depression and anxiety symptoms and keeping stable throughout the entire sailing. The Low-Inverted U group showed a moderate growth during the first half of the sailing, and then a moderate decrease during the second half of the sailing for both depression and anxiety symptoms. The mean level of depression symptoms reached its peak value (31.19) at the midpoint (Day 28 of the 55-day sailing) based on the estimated results, while the mean level of anxiety symptoms reached its peak value (40.44) at the midpoint (Day 27 of the 55-day sailing). Throughout the entire sailing, depression and anxiety symptoms of the sailors in the Low-Inverted U group stayed at a low level,especially the level of depression symptoms. The Moderate-Deterioration group was characterized by a moderate initial level of depression and anxiety symptoms but a linear growth throughout the entire sailing. The predicted level of depression (48.15) at the end of sailing were close to the cut-off points of SDS.

Table 8 presented the longitudinal association between baseline hardiness and the joint trajectories of depression and anxiety symptoms, controlling sociodemographic variables. The Low-Inverted U group was set as reference. The results of multinomial logistic regression showed that the sailors with higher level of baseline hardiness were less likely to belong to the Moderate-Deterioration group (*OR* = 0.87 [0.82, 0.92], *p* < 0.001) and High Stable group (*OR* = 0.76 [0.69, 0.83], *p* < 0.001). In addition, less education years (*OR* = 2.18 [1.39, 3.42], *p* < 0.001) were associated with lower likelihood of belonging to the Moderate-Deterioration group. Besides, the sailors with less education years (*OR* = 1.73 [1.29, 2.32], *p* < 0.001) were also less likely to belong to the High-Stable group.

**Table 8:** The predictive role of hardiness on joint trajectories of depression and anxiety symptoms

	*Moderate-Deterioration*		*High-Stable*	
	*B* (*SE*)	*OR* [95% CI]	*B* (*SE*)	*OR* [95% CI]
Married (Other as reference)	-0.41 (0.41)	0.66 [0.29, 1.48]	-0.69 (0.43)	0.50 [0.22, 1.17]
Only child (non-single as reference)	0.49 (0.50)	1.63 [0.61, 4.34]	-0.12 (0.31)	0.89 [0.48, 1.63]
Age	-0.13 (0.14)	0.89 [0.66, 1.15]	-0.12 (0.09)	0.89 [0.74, 1.05]
Education	0.78 (0.23)	2.18 [1.39, 3.42]^***^	0.55 (0.15)	1.73 [1.29, 2.32]^***^
Shift work exposure	-0.22 (0.19)	0.80 [0.55, 1.16]	0.11 (0.08)	1.11 [0.95, 1.31]
Hardiness	-0.14 (0.03)	0.87 [0.82, 0.92]^***^	-0.28 (0.05)	0.76 [0.69, 0.83]^***^

Low-Inverted U group as reference. Hardiness was standardized before included in the logistic regression model

^*^ *p* < .05. ^**^ *p* < .01. ^***^ *p* < .001

## Discussion

In the present study, we investigated the independent and joint trajectories of depression and anxiety symptoms of Chinese sailors on 18-h watchstanding schedule and the predictive role of hardiness on these trajectories during a 55-day sailing. The results generally supported our hypotheses: (1) there existed different groups of sailors with different independent and joint trajectories of depression and anxiety symptoms. Specifically, there were 2 different groups for the independent trajectories of depression (High-Deterioration group and Low-Inverted U group), 3 for the independent trajectories of anxiety (High-Stable group, Low-Rapid Deterioration group, and Moderate-Inverted U group), and 3 for the joint trajectories of depression and anxiety (Low-Inverted U group, Moderate-Deterioration group, and High-Stable group). (2) The baseline level of hardiness could predict these trajectories controlled for sociodempgraphic variables. The sailors with higher level of baseline hardiness were more likely to belong to more mentally healthy trajectories (e.g., Low-Inverted U group for depression, Moderate-Inverted U group for anxiety, Low-Inverted U group for the joint trajectories of depression and anxiety). (3) Some sociodemographic variables were associated with these trajectories. For example, the sailors with higher educational level were more likely to have less healthy trajectories (e.g., Low-Rapid Deterioration group for anxiety and High-Stable group for joint trajectories of depression and anxiety).

### Independent and joint trajectories of depression and anxiety symptoms

The independent trajectories of depression and anxiety symptoms both followed an inverted-U changing trend at the population level. The results of LGMM also showed that depression and anxiety symptoms of more than 70% of the sailors followed the inverted-U changing trend (a moderate growth during the first half of the sailing, and then a moderate decrease during the second half of the sailing for both depression and anxiety symptoms), although there were signifcant heterogeneity in these trajectories. In the study field of isolated, confined, and extreme environments (ICEs), this changing trend is called “third quarter phenomenon” [51]. This phenomemon was first reported in a polar mission that there was a significant deterioration of emotional states among Antarctic expeditioner during the third quarter of the polar mission and a slight recover at the end of mission [51]. Then, the third quarter phenomenon was also found in other ICEs such as spaceflight or submarine [52]. The cabin environment in the ships during the sailing, which was employed as the study context in the present study, can also be treated as an isolated and closed environment. There is currently no unified explanation for the mechanism of third quarter phenomenon. The increase of negative emotions may be resulted from higher arousal during the third-quarter period of the mission. [53]. Another explanation is based on the stress theory that the peak of negative emotions of sailors reflects that they are in the exhaustion phase of stress [54]. The authors suggested that the inverted-U changing trend reflected the process of adaptation to non-24-h work shifts among sailors according to the job demands-resources model [27, 28]. In the first half of sailing, sailors consumed their personal job resources to meet the challenging job demands on the non-24-h-shift-work condition. In this process, job demands exceeded job resources which lead to negative outcomes such as depression and anxiety symptoms [27]. In the second half of sailing, the sailors gradually adapted to to the special working condition, and the anticipation of returning home significantly enhanced their work motivation. The gradually formed balance between job demands and job resources contribute to the decrease of depression and anxiety symptoms [55].

Recently, some researchers argued that the third quarter phenomenon is not a typical occurance in ICEs [51, 56]. For example, a recent meta-analysis of studies on time-dependent mood fluctuations in Antarctic personnel did not support the existence of third quarter phenomenon [56]. Similarly, we cannot draw a conclusion that there exists third quarter phenomenon during a prolonged period of non-24-h working schedules based on evidence of the present study. More studies should be conducted to figure out the trajectories of depression and anxiety among shift workers on non-24-h shift working schedules.

### The predictive role of hardiness on the trajectories of depression and anxiety symptoms

The present study found that hardiness could predict the trajectories of depression and anxiety symptoms during a prolonged period of non-24-h shift work. Participants with higher level of hardiness were more likely to belong to more healthy group (e.g., Low-Inverted U group for joint trajectories of depression and anxiety). The results were consistent with previous studies on shift work tolerance [22, 23]. Hardiness is a personality trait that is defined by a high sense of life and work commitment, high belief in control, and high openness to change and challenges, as well as a more positive perception of stress [24]. From the view of job demands-resources model, hardiness can be regarded as a kind of personal resources [27]. Participants with higher level of hardiness had more job resources, therefore they were less likely to experience obvious mental health problems when facing strict job demands like working on a non-24-h shift schedule for 2 months. Our findings further confirmed that hardiness is a psychological predictor of shift work tolerance [22, 23].

### Strengths and pratical implications

The present study has some strengths and practical implications [30]. First, we employed a longitudinal design and focused on the short-term effect of non-24-h working schedules on mental health status of shift workers during a prolonged continuous period of non-24-h schedules (about 2 months).The shift workers on non-24-h working schedules, such as sailors and submariners, are a special population. Unlike general shift workers, most of shift workers on non-24-h working schedules have to work for a longer period of time (more than 2 months) without leave. Thus, the mental health status of shift workers during a prolonged continuous period of non-24-h schedules are of great research value. However, previous longitudinal studies in the field of shift work tolerance paid more attention to long term effect of shift work on shift workers. For example, Thun et al. [57] conducted a 2-year longitudinal study on the effect of night shift on depression and anxiety symptoms of nurses. Saksvik-Lehouillier et al. [26] also investigated the changes of depression and anxiety symptoms of shift-working nurses over 2 years. These studies are unable to clarify changing trend of mental status during the period of non-24-h rotating shift schedule.

Second, we set 5 measurement time points throughout the entire sailing which covered all stages of the sailing. Based on such intensive measurements, we were able to provide a more comprehensive depiction of the changing trends of depression and anxiety symptoms among sailors during the sailing, which cannot be found in previous studies of pre-post design (only had 2 measurement time points). For example, a former study found that there were no significant changes between mood states of submariners measured at Day 21 and Day 51 during a 70-day patrol mission at sea [8]. Such results may lead the authors and readers to draw a wrong conclusion that a 70-day sailing will not affect the emotions of submariners. Although we also found that there were no significant differences between the depression and anxiety symptoms measured at Day 14 and Day 42 during a 55-day sailing, the whole trajectories of depression and anxiety symptoms were in the shape of an inverted U in this study. Thus, the actual situation may be a growth of depression and anxiety symptoms in the first half of the sailing followed by a decrease during the second half, resulting in an illusion of no change having occurred at all. The complete changing trends of depression and anxiety throughout the sailing provides valuable insights into the timely prevention and intervention of mental health problems during a prolonged non-24-h shift working schedule [30].

Third, we employed a person-centered approach (e.g., LGMM) to identify individual differences of the trajectories of depression and anxiety during a prolonged period of non-24-h shift working schedule. To our best knowledge, no previous studies investigated the distinct subgroups in the trajectories of depression and anxiety symptoms with different baseline levels and changing trends among shift workers [30]. What is more, we also demonstrated the predictive and protecting values of hardiness on mental health of shift workers. These results suggested that hardiness is a potential predictor in personnel selection of sailors, submariners, and other shift workers on non-24-h working schedules. Besides, hardiness has been proved to be a malleable construct that can be improved by many types of intervention programs [30, 58, 59]. Thus, the hardiness-based intervention programs should be encouraged among shift workers to protect their mental health.

### Limitations and future research

Although our study had some strengths, several limitations should also be acknowledged, which can be addressed in future research. First, this study was conducted among the Chinese sailors on 18-h watchstanding schedule, thus the results may be unable to be replicated in other shift workers. More studies should be performed to investigate the trajectories of depression and anxiety among shift workers on other shift-working schedule or other working environment. Second, this study did not recruit female participants due to the rareness of female sailors in China. Third, The use of forced answering might have impacted the answering [60]. Fourth, the present study only retrospectively measured the average levels of depression and anxiety symptoms over a period of time (2 weeks). However, it is worth examining emotion fluctuation during different parts of the day/night or different parts of shift work schedule (work, rest, or leisure time), which should be conducted in the future research. Finally, the present study only measured the levels of depression and anxiety symptoms during the sailing, but did not track the changing trends after the sailing. Future studies can investigate the changing trends of depresison and anxiety symptoms after a prolonged period of non-24-h shift working schedule, which can further clarify how long will it take for a shift worker to totally recover from the prolonged non-24-h working schedule or whether there exist any long-term cumulative effects of a prolonged period of non-24-h shift working schedule that cannot be fully recovered from.

## Conclusion

During a prolonged period of non-24-h shift working schedule, depression and anxiety symptoms of the whole group of shift workers presented an inverted-U changing trend. There were significant individual differences in the independent and joint trajectories of depression and anxiety symptoms among shift workers. In concrete terms, the majority of sailors showed an inverted-U-shaped changing trends in anxiety and depression, and the absolute levels of anxiety and depression remain at low or moderate levels throughout the entire sailing. At the same time, a minority of sailors showed less mentally healthy trajectories: (1) some sailors experienced significant deterioration in anxiety and depression during the sailing; (2) others had a high level of depression and anxiety at baseline and keep stable with high levels throughout the entire sailing. Hardiness may be a predictive and protecting factor that the sailors with higher levels of hardiness were more likely to follow a more healthy mental health pattern during the prolonged period of non-24-h shift working schedule. Thus, we encourage the implementation of hardiness-based intervention programs among the shift workers on non-24-h working and rest schedules.

## Data Availability

All data generated or analysed during this study are available from the corresponding author on reasonable request.

## Abbreviations

ANOVA: Analysis of Variance
C-DRS-15: Chinese version of 15-item Dispositional Resilience Scale
SDS: Zung Self-rating Depression Scale
SAS: Zung Self-rating Anxiety Scale
LGMM: Latent growth mixture models
LCGA: Latent curve growth analysis
TLI: Tucker-Lewis index
CFI: Comparative fit index
RMSEA: Root mean square error of approximation
SRMR: Standardized root mean square residual
BIC: Bayesian information criteria
aBIC: Sample-size adjusted Bayesian information criterion
AIC: Aikaike information criterion
LRT: Lo-Mendell-Rubin likelihood ratio test
BLRT: Bootstrap likelihood ratio test
CI: Confdence interval
PPLGMM: Parallel process lantent growth mixture models
I: Intercept
S: Slope
Q: Quadratic
OR: Odds ratio
ICEs: Isolated, confined, and extreme environments
